# An analog-sensitive allele of Aurora kinase B is lethal in mouse

**DOI:** 10.17912/micropub.biology.000491

**Published:** 2021-11-23

**Authors:** Berta N. Vazquez, Suzanne M. Quartuccio, Karen Schindler

**Affiliations:** 1 Chromatin Biology Laboratory, Josep Carreras Leukaemia Research Institute (IJC), Badalona, Barcelona, Spain; 2 Department of Genetics, Rutgers University, Piscataway, NJ, USA; 3 Departament de Biologia Cellular, Fisiologia i Immunologia, Universitat Autònoma de Barcelona (UAB), Barcelona, Spain; 4 Department of Biological Sciences, Seton Hall University, South Orange, NJ, USA

## Abstract

The mammalian genome encodes three Aurora protein kinase homologs (AURKA/B/C) which regulate chromosome segregation in nearly every cell type. AURKC expression is largely limited to meiotic cells. Because of the similarity in sequences between AURKB and AURKC, determining their separate functions during meiosis is challenging. We designed a chemical genetics approach to investigate AURKB function. Using Crispr/Cas9 genome editing in mouse, we replaced an ATP binding pocket amino acid to permit binding of cell-permeable ATP analogs. We also introduced a second site supressor mutation to tolerate the pocket enlargement. Heterozygous mice were fertile, but never produced homozygous analog-sensitive mice. Because *Aurkb* is an essential gene, we conclude that this analog-sensitive allele is either catalytically inactive or not fully catalytically active in mouse.

**Figure 1:**
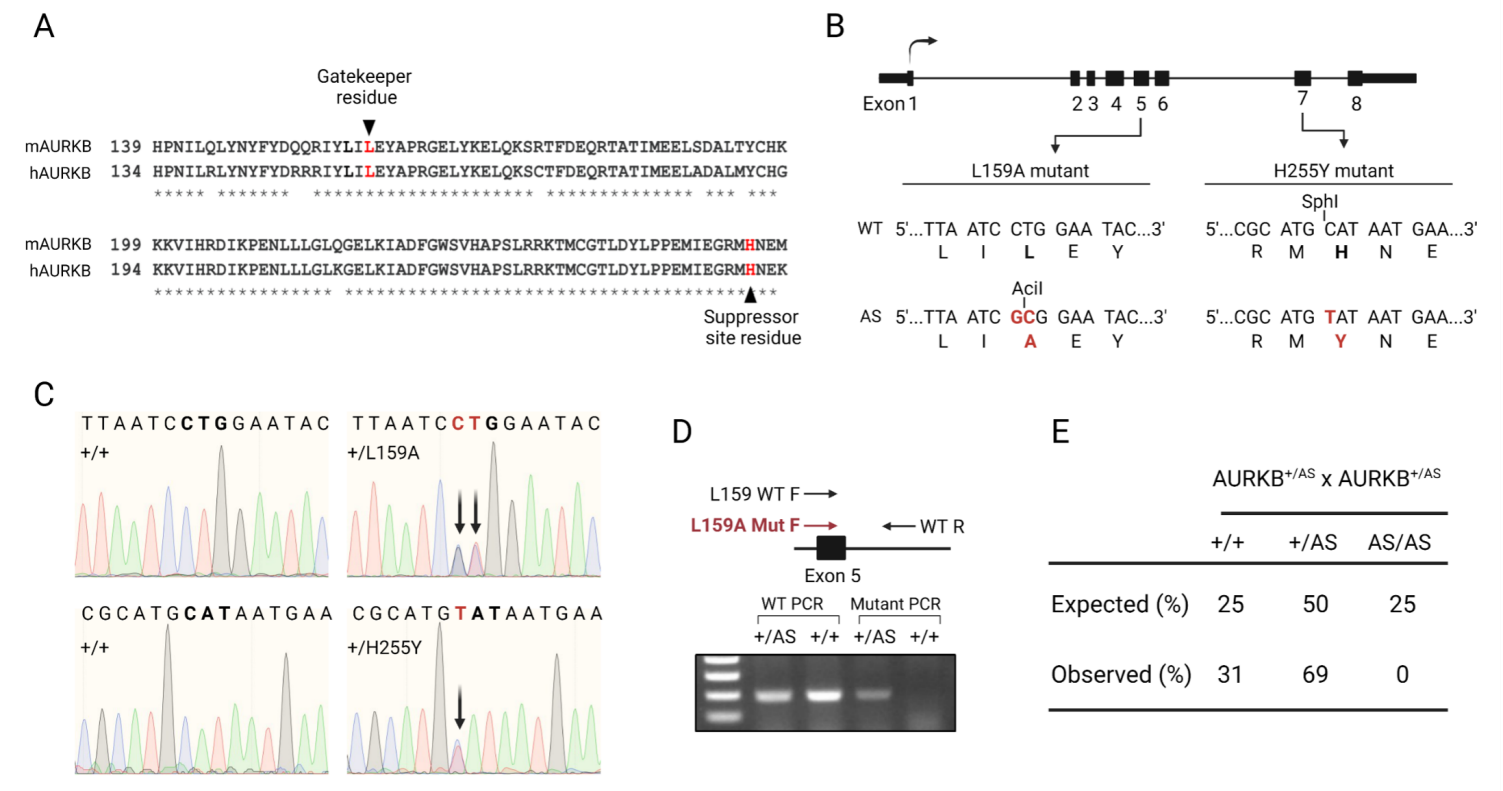
**Generation of analog sensitive AURKB mutant mice**. **A)** Partial protein sequence alignment of mouse (m) and human (h) Aurora B kinases. Gatekeeper and suppressor site residues are highlighted in red. Asterisks below sequences denote residue conservation. **B)** AURKB gene organization together with partial DNA and protein sequences of WT and analog sensitive mutant alleles. Restriction sites used for diagnosis are indicated above DNA sequences (AciI for L159 to A substitution and SphI for H255 to Y substitution). **C)** Sanger sequencing confirming expected DNA substitutions. **D)** Scheme of genotyping strategy and representative PCR result image for L159 WT and mutant allele. **E)**Mice heterozygous for the AURKB-AS allele were intercrossed and the progeny genotyped. A total number of 80 pups coming from 8 different crosses were analyzed. The proportion of mutant mice was compared to expected Mendelian ratios (25%, 50%, 25%) and statistical analysis was conducted with a chi-square test (*P*-value < 0.001 by χ^2^).

## Description

The Aurora protein kinases (AURK) are essential regulators of chromosome segregation in mitotic and meiotic cell divisions (Carmena and Earnshaw 2003). Unlike mitotically dividing cells which express two AURKs (AURKA and AURKB), mammalian oocytes, which undergo meiosis, express three AURK homologs: AURKA/B/C (Brown *et al.* 2004; Nguyen *et al.* 2018). Because AURKC shares high sequence homology with AURKB, standard approaches such as RNAi knockdown (Chen *et al.* 2005; Sharif *et al.* 2010) and small molecule inhibition (Chen *et al.* 2005; Lane *et al.* 2010; Sharif *et al.* 2010; Shuda *et al.* 2009; Swain *et al.* 2008) to understand their roles do not provide the specificity required to assess non-overlapping functions. Furthermore, these kinases can compensate for one another in oocytes from mouse knockout strains (Balboula and Schindler 2014; Fernandez-Miranda *et al.* 2011; Nguyen *et al.* 2018; Schindler *et al.* 2012), making phenotype assessments less clear. Therefore, alternative strategies to provide specificity are needed.

Chemical genetics is a strategy that documents superior specificity in inhibiting protein kinases. This approach involves enlarging the kinase ATP-binding pocket by introduction of an amino acid substitution. This enlargement allows specific acceptance of cell-permeable, bulky ATP analog inhibitors that cannot bind other kinases in the cell (Garske *et al.* 2011). Although most protein kinases tolerate the ATP pocket enlargement, approximately 30% of protein kinases do not (Zhang *et al.* 2005). A previous report demonstrates that human AURKB ATP-pocket enlargement (Leucine 154 to Alanine) abolishes its catalytic activity. Excitingly, this activity can be rescued by mutation of a second site suppressor in the C-terminus of the kinase (Histidine 250 to Tyrosine). When HeLa and U2OS cells were transiently transfected with the analog sensitive human *AURKB* allele, the kinase was active and cell division was not altered (Hengeveld *et al.* 2012). Based on these data, we were keen to adopt this approach to study the potential unique functions of AURKB. We used Crispr/Cas9 genome editing to introduce the ATP-binding pocket point mutation (L159 in mouse) and second site suppressor mutation (H255 in mouse) into the mouse genome (Fig. 1A). After confirmation by restriction digestion (Fig. 1B) and Sanger sequencing (Fig. 1C), we obtained 4 founders (2 male, 2 female) that were heterozygous for the analog-sensitive allele on the same chromosome. To expand the colony, we set up breeding cages using the heterozygous founders. Heterozygous animals were fertile and gave birth to pups. We also crossed heterozygous pups to increase chances of obtaining homozygous animals. After genotyping 80 pups that were born to 8 heterozygous mating pairs, we did not obtain any homozygous mice; wild-type and heterozygotes were born at altered Mendelian ratios (Fig. 1D-E). Because *Aurkb* is an essential gene (Fernández-Miranda *et al.* 2011), these data indicate that this analog-sensitive allele of *Aurkb* is either catalytically inactive or not fully catalytically active in mouse and that it cannot be used to determine specific AURKB functions in mouse oocytes.

## Reagents

C57BL/6J mice: Jackson Laboratory

Cas9 IDT

L-A RNA guide (PAM sequence in parentheses) from Sigma: ACTACTTCTACGACCAGCAG (AGG)

L-A donor oligo (mutations in lower case): CAGGCACTAGATCCCACACCAGGTAGGCCCTGACCGTGGCAGTCCGCTGCTCATCGAAGGTCCGACTCTTCTGCAGT TCCTTGTAGAGTTCCCCGCGAGGGGCGTATTCCgcGATTAAGTAGATtCTCTGCTGGTCGTAGAAGTAGTTGTAGAG TTGAAGGATGTTGGGATGTCTGG

H-Y RNA guide (PAM sequence in parentheses) from Sigma: TGCCCCCAGAGATGATTGAG (GGG)

H-Y donor oligo (mutations in lower case): TCCGACGATACGTCTCACTGTGGCTAGGGCTCTCGAAGGGTGGGTTCCCCACCATCAGTTCATAGCAGAGCACCCC GATGCACCATAGATCTACCATTTCATTATaCATGCGgCCCTCAATCATCTCTGGGGGCAGATAGTCCAGCGTGCCG CACATGGTCTTCCTCCTGGTGGGATGAG

Genotyping Primers:

**L159A substitution**

L159WT F: 5’- CCAGCAGAGGATCTACTTAATCCT -3’

L159AS F: 5’- CCAGCAGAGGATCTACTTAATCGC -3’

WT R: 5’- ATGATctgtgagggtccacacaag -3’

**H255Y substitution**

H255WT F: 5’- GAGATGATTGAGGGGCGCATGCAT -3’

H255Y F: 5’- GAGATGATTGAGGGGCGCATGTAT -3’

WT R: 5’- CTGGCTCCTACAGTACACAAAGG -3’
